# 3-Ethyl-5-(4-meth­oxy­phen­oxy)-2-(pyridin-4-yl)-3*H*-imidazo[4,5-*b*]pyridine

**DOI:** 10.1107/S1600536811023543

**Published:** 2011-06-22

**Authors:** S. Ranjith, A. SubbiahPandi, A. D. Suresh, K. Pitchumani

**Affiliations:** a Department of Physics, Presidency College (Autonomous), Chennai 600 005, India; b School of Chemistry, Madurai Kamaraj University, Madurai 625 021, India

## Abstract

In the title compound, C_20_H_18_N_4_O_2_, the imidazopyridine fused ring system is almost perpendicular to the benzene ring [dihedral angle = 87.6 (5)°]. The pyridine ring makes a dihedral angle of 35.5 (5)° with the mean plane of the imidazopyridine fragment. The crystal structure is stabilized by an aromatic π–π stacking inter­action between the phenyl rings of neighbouring mol­ecules [centroid–centroid distance = 3.772 (2) Å, inter­planar distance = 3.546 (2) Å and slippage = 1.286 (2) Å].

## Related literature

For the biological activity of pyridine derivatives, see: Passannanti *et al.* (1998 ▶); Jiyeon *et al.* (2010 ▶); Abdel-Alim *et al.* (2005 ▶); Girgis *et al.* (2006 ▶); Slominska *et al.* (2008 ▶); Spanka *et al.* (2010 ▶). For a related structure, see: Ouzidan *et al.* (2010 ▶). For *sp* ^3^ hybridization, see: Beddoes *et al.* (1986 ▶).
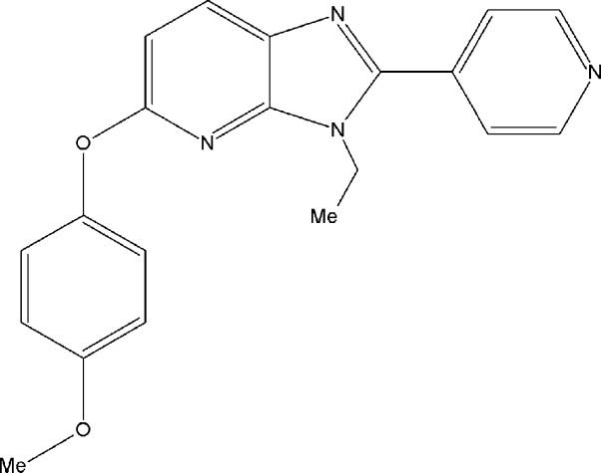


## Experimental

### 

#### Crystal data

C_20_H_18_N_4_O_2_
*M* *_r_* = 346.38
Monoclinic, 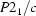

*a* = 13.6591 (4) Å
*b* = 13.7104 (4) Å
*c* = 9.3177 (2) Å
β = 98.940 (1)°
*V* = 1723.74 (8) Å^3^
*Z* = 4
Mo *K*α radiation
μ = 0.09 mm^−1^
*T* = 293 K
0.25 × 0.22 × 0.19 mm

#### Data collection

Bruker APEXII CCD area-detector diffractometer
Absorption correction: multi-scan (*SADABS*; Sheldrick, 1996 ▶) *T*_min_ = 0.981, *T*_max_ = 0.985
24827 measured reflections
5887 independent reflections
3938 reflections with *I* > 2σ(*I*)
*R*_int_ = 0.027

#### Refinement

*R*[*F* ^2^ > 2σ(*F* ^2^)] = 0.046
*wR*(*F* ^2^) = 0.140
*S* = 1.00
5887 reflections
237 parameters
H-atom parameters constrained
Δρ_max_ = 0.30 e Å^−3^
Δρ_min_ = −0.20 e Å^−3^

### 

Data collection: *APEX2* (Bruker, 2004 ▶); cell refinement: *SAINT* (Bruker, 2004 ▶); data reduction: *SAINT*; program(s) used to solve structure: *SHELXS97* (Sheldrick, 2008 ▶); program(s) used to refine structure: *SHELXL97* (Sheldrick, 2008 ▶); molecular graphics: *ORTEP-3* (Farrugia, 1997 ▶); software used to prepare material for publication: *SHELXL97* and *PLATON* (Spek, 2009 ▶).

## Supplementary Material

Crystal structure: contains datablock(s) global, I. DOI: 10.1107/S1600536811023543/lx2189sup1.cif

Structure factors: contains datablock(s) I. DOI: 10.1107/S1600536811023543/lx2189Isup2.hkl

Supplementary material file. DOI: 10.1107/S1600536811023543/lx2189Isup3.cml

Additional supplementary materials:  crystallographic information; 3D view; checkCIF report

## Figures and Tables

**Fig. 1.**
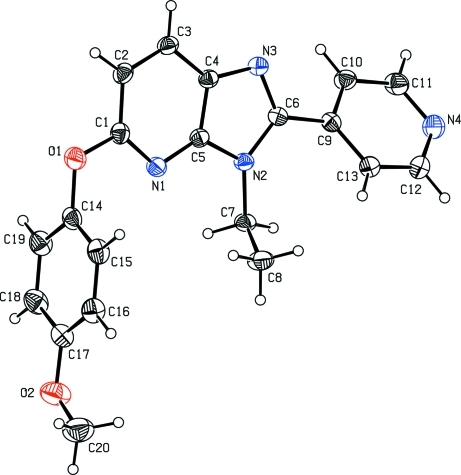
The structure of showing the atom-numbering scheme. The displacement ellipsoids are drawn at the 30% probability level.

## supplementary crystallographic information

### Comment

Pyridine derivatives has numerous applications in medicinal chemistry (Passannanti *et al.*, 1998). Furthermore, the imidazo[4,5-*b*]pyridine moiety is also an important heterocyclic nucleus which has been used extensively in medicinal chemistry. In fact, the heterocycles derived from these intermediates have been tested for their potential as anti-neuroinflammatory (Jiyeon *et al.*, 2010). Pyridine-3-carboxamides have gained attention because of their diverse pharmacological properties such as anti-inflammatory (Abdel-Alim *et al.*, 2005), anticancer (Girgis *et al.*, 2006), cytoprotective (Slominska *et al.*, 2008), and anxiolytic (Spanka *et al.*, 2010) activities. Against this background, and in order to obtain detailed information on molecular conformations in the solid state, an X-ray study of the title compound was carried out.

The bond lengths and angles in (Fig. 1) agree with those observed in other imidazopyridine derivatives (Ouzidan *et al.*, 2010). The imidazopyridine ring system is essentially planar, with maximum deviation of 0.013 (1)° for atom C1. The sum of bond angles around N2[359.2 (9)°] of the imidazole ring is in accordance with *sp*^3^ hybridization (Beddoes *et al.*, 1986). The imidazopyridine ring system makes dihedral angles of 35.5 (5) and 87.6 (5)°, respectively, with the pyridine and phenyl rings and also the dihedral angle between the pyridine and phenyl ring is 87.0 (6)°. It shows that the phenyl ring is perpendicular to both the imidazopyridine and pyridine rings. The atoms O1 and O2 are deviated by 0.039 (1) and - 0.021 (1)Å from the leastsquares plane of the phenyl ring.

The molecules lack hydrogen bonding functionality and pack in layers parallel to the (100) planes. The crystal structure is stabilized by an aromatic π–π stacking interaction between the phenyl rings of adjacent molecules, with a Cg···Cg distance  of 3.772 (2) Å and an interplanar distance of 3.546 (3) Å resulting in a slippage of 1.286 (3) Å (Cg  is the centroid of the C14–C19 phenyl ring).

### Experimental

*N*-ethyl-6-(4-methoxyphenoxy)pyridin-2-amine (0.23 g, 1 mmol) and amide (0.12 g, 1 mmol) successively added to Al^3+^–Y in xylene at 145°C. After stirring for 16 h, the mixture was diluted with dichloromethane. After removing the catalyst by filtration, followed by solvent evaporation, the resulting crude product was finally purified by column chromatography (silica gel). Single crystals suitable for X-ray diffraction were obtained by slow evaporation of a solution of the title compound in ethylacetate at room temperature.

### Refinement

All H atoms were fixed geometrically and allowed to ride on their parent C atoms, with C—H distances fixed in the range 0.93–0.97 Å with *U*_iso_(H) = 1.5*U*_eq_(C) for methyl H 1.2*U*_eq_(C) for other H atoms.

### Crystal data

**Table:**

C_20_H_18_N_4_O_2_	*F*(000) = 728
*M**_r_* = 346.38	*D*_x_ = 1.335 Mg m^−^^3^
Monoclinic, *P*2_1_/*c*	Mo *K*α radiation, λ = 0.71073 Å
Hall symbol: -P 2ybc	Cell parameters from 5887 reflections
*a* = 13.6591 (4) Å	θ = 1.5–32.0°
*b* = 13.7104 (4) Å	µ = 0.09 mm^−^^1^
*c* = 9.3177 (2) Å	*T* = 293 K
β = 98.940 (1)°	Block, white crystalline
*V* = 1723.74 (8) Å^3^	0.25 × 0.22 × 0.19 mm
*Z* = 4	

### Data collection

**Table:**

Bruker APEXII CCD area-detector diffractometer	5887 independent reflections
Radiation source: fine-focus sealed tube	3938 reflections with *I* > 2σ(*I*)
graphite	*R*_int_ = 0.027
Detector resolution: 10.0 pixels mm^-1^	θ_max_ = 32.1°, θ_min_ = 1.5°
ω and φ scans	*h* = −20→20
Absorption correction: multi-scan (*SADABS*; Sheldrick, 1996)	*k* = −20→20
*T*_min_ = 0.981, *T*_max_ = 0.985	*l* = −13→12
24827 measured reflections	

### Refinement

**Table:**

Refinement on *F*^2^	Primary atom site location: structure-invariant direct methods
Least-squares matrix: full	Secondary atom site location: difference Fourier map
*R*[*F*^2^ > 2σ(*F*^2^)] = 0.046	Hydrogen site location: difference Fourier map
*wR*(*F*^2^) = 0.140	H-atom parameters constrained
*S* = 1.00	*w* = 1/[σ^2^(*F*_o_^2^) + (0.068*P*)^2^ + 0.2334*P*] where *P* = (*F*_o_^2^ + 2*F*_c_^2^)/3
5887 reflections	(Δ/σ)_max_ < 0.001
237 parameters	Δρ_max_ = 0.30 e Å^−^^3^
0 restraints	Δρ_min_ = −0.20 e Å^−^^3^

### Special details

**Table:**

Geometry. All e.s.d.'s (except the e.s.d. in the dihedral angle between two l.s. planes) are estimated using the full covariance matrix. The cell e.s.d.'s are taken into account individually in the estimation of e.s.d.'s in distances, angles and torsion angles; correlations between e.s.d.'s in cell parameters are only used when they are defined by crystal symmetry. An approximate (isotropic) treatment of cell e.s.d.'s is used for estimating e.s.d.'s involving l.s. planes.
Refinement. Refinement of *F*^2^ against ALL reflections. The weighted *R*-factor *wR* and goodness of fit *S* are based on *F*^2^, conventional *R*-factors *R* are based on *F*, with *F* set to zero for negative *F*^2^. The threshold expression of *F*^2^ > σ(*F*^2^) is used only for calculating *R*-factors(gt) *etc*. and is not relevant to the choice of reflections for refinement. *R*-factors based on *F*^2^ are statistically about twice as large as those based on *F*, and *R*- factors based on ALL data will be even larger.

### Fractional atomic coordinates and isotropic or equivalent isotropic displacement parameters (Å^2^)

**Table:**

	*x*	*y*	*z*	*U*_iso_*/*U*_eq_	
C1	0.34424 (9)	0.10472 (9)	0.42615 (13)	0.0383 (3)	
C2	0.34457 (9)	0.18018 (9)	0.52777 (14)	0.0406 (3)	
H2	0.3850	0.2344	0.5240	0.049*	
C3	0.28465 (9)	0.17335 (9)	0.63296 (13)	0.0389 (3)	
H3	0.2834	0.2222	0.7020	0.047*	
C4	0.22585 (8)	0.09028 (8)	0.63192 (12)	0.0335 (2)	
C5	0.23419 (8)	0.02078 (8)	0.52553 (12)	0.0330 (2)	
C6	0.12671 (8)	−0.02604 (8)	0.66320 (12)	0.0332 (2)	
C7	0.16889 (10)	−0.14643 (8)	0.46766 (13)	0.0403 (3)	
H7A	0.1356	−0.1952	0.5182	0.048*	
H7B	0.2363	−0.1683	0.4670	0.048*	
C8	0.11655 (12)	−0.13785 (11)	0.31346 (15)	0.0547 (4)	
H8A	0.0490	−0.1186	0.3137	0.082*	
H8B	0.1181	−0.1997	0.2655	0.082*	
H8C	0.1493	−0.0897	0.2629	0.082*	
C9	0.05197 (8)	−0.08407 (8)	0.72257 (12)	0.0341 (2)	
C10	0.05089 (10)	−0.08248 (9)	0.87147 (13)	0.0427 (3)	
H10	0.0986	−0.0476	0.9329	0.051*	
C11	−0.02193 (11)	−0.13343 (10)	0.92665 (15)	0.0500 (3)	
H11	−0.0225	−0.1305	1.0262	0.060*	
C12	−0.08936 (10)	−0.18810 (10)	0.70402 (15)	0.0459 (3)	
H12	−0.1370	−0.2251	0.6457	0.055*	
C13	−0.02073 (9)	−0.13820 (9)	0.63755 (13)	0.0386 (3)	
H13	−0.0233	−0.1409	0.5373	0.046*	
C14	0.40351 (9)	0.04175 (9)	0.22118 (14)	0.0425 (3)	
C15	0.33767 (10)	0.04307 (10)	0.09494 (15)	0.0488 (3)	
H15	0.2913	0.0930	0.0769	0.059*	
C16	0.34062 (10)	−0.03057 (11)	−0.00606 (15)	0.0508 (3)	
H16	0.2964	−0.0299	−0.0927	0.061*	
C17	0.40900 (10)	−0.10480 (10)	0.02181 (15)	0.0479 (3)	
C18	0.47462 (11)	−0.10467 (11)	0.14910 (16)	0.0546 (4)	
H18	0.5211	−0.1544	0.1679	0.066*	
C19	0.47204 (10)	−0.03162 (11)	0.24862 (16)	0.0512 (3)	
H19	0.5167	−0.0318	0.3348	0.061*	
C20	0.35584 (16)	−0.18189 (16)	−0.2055 (2)	0.0799 (5)	
H20A	0.2878	−0.1767	−0.1920	0.120*	
H20B	0.3653	−0.2416	−0.2555	0.120*	
H20C	0.3727	−0.1276	−0.2621	0.120*	
N1	0.29165 (7)	0.02406 (7)	0.42238 (11)	0.0374 (2)	
N2	0.17080 (7)	−0.05379 (7)	0.54614 (10)	0.0340 (2)	
N3	0.15774 (7)	0.05932 (7)	0.71772 (10)	0.0357 (2)	
N4	−0.09155 (9)	−0.18655 (9)	0.84645 (13)	0.0512 (3)	
O1	0.40353 (7)	0.11775 (7)	0.32231 (11)	0.0511 (2)	
O2	0.41700 (9)	−0.18162 (9)	−0.06945 (12)	0.0716 (3)	

### Atomic displacement parameters (Å^2^)

**Table:**

	*U*^11^	*U*^22^	*U*^33^	*U*^12^	*U*^13^	*U*^23^
C1	0.0354 (6)	0.0366 (6)	0.0433 (6)	−0.0005 (4)	0.0079 (5)	0.0034 (5)
C2	0.0382 (6)	0.0315 (6)	0.0517 (7)	−0.0044 (5)	0.0052 (5)	−0.0009 (5)
C3	0.0407 (6)	0.0313 (5)	0.0433 (6)	−0.0008 (5)	0.0022 (5)	−0.0056 (5)
C4	0.0353 (5)	0.0304 (5)	0.0338 (5)	0.0009 (4)	0.0027 (4)	−0.0008 (4)
C5	0.0350 (5)	0.0299 (5)	0.0338 (5)	0.0000 (4)	0.0042 (4)	0.0004 (4)
C6	0.0364 (5)	0.0316 (5)	0.0318 (5)	0.0020 (4)	0.0057 (4)	0.0008 (4)
C7	0.0471 (7)	0.0287 (5)	0.0468 (7)	−0.0017 (5)	0.0128 (5)	−0.0070 (5)
C8	0.0681 (9)	0.0502 (8)	0.0454 (7)	−0.0077 (7)	0.0075 (7)	−0.0135 (6)
C9	0.0375 (6)	0.0297 (5)	0.0360 (5)	0.0038 (4)	0.0084 (4)	0.0031 (4)
C10	0.0508 (7)	0.0416 (6)	0.0365 (6)	−0.0059 (5)	0.0091 (5)	−0.0021 (5)
C11	0.0647 (9)	0.0483 (7)	0.0403 (7)	−0.0071 (6)	0.0187 (6)	0.0014 (6)
C12	0.0408 (7)	0.0469 (7)	0.0496 (7)	−0.0049 (5)	0.0064 (5)	0.0004 (6)
C13	0.0394 (6)	0.0402 (6)	0.0357 (6)	0.0012 (5)	0.0044 (5)	0.0025 (5)
C14	0.0419 (6)	0.0421 (6)	0.0470 (7)	−0.0027 (5)	0.0181 (5)	0.0038 (5)
C15	0.0454 (7)	0.0443 (7)	0.0571 (8)	0.0077 (6)	0.0097 (6)	0.0095 (6)
C16	0.0489 (7)	0.0559 (8)	0.0462 (7)	0.0039 (6)	0.0025 (6)	0.0070 (6)
C17	0.0490 (7)	0.0491 (7)	0.0473 (7)	0.0052 (6)	0.0129 (6)	0.0019 (6)
C18	0.0499 (8)	0.0579 (9)	0.0554 (8)	0.0177 (7)	0.0060 (6)	0.0030 (7)
C19	0.0466 (7)	0.0594 (9)	0.0468 (7)	0.0080 (6)	0.0045 (6)	0.0042 (6)
C20	0.0881 (13)	0.0854 (13)	0.0627 (11)	0.0002 (11)	0.0005 (9)	−0.0198 (10)
N1	0.0399 (5)	0.0342 (5)	0.0396 (5)	−0.0011 (4)	0.0109 (4)	−0.0010 (4)
N2	0.0402 (5)	0.0285 (4)	0.0342 (5)	−0.0025 (4)	0.0085 (4)	−0.0030 (4)
N3	0.0396 (5)	0.0329 (5)	0.0346 (5)	0.0001 (4)	0.0060 (4)	−0.0017 (4)
N4	0.0538 (7)	0.0511 (7)	0.0517 (7)	−0.0089 (5)	0.0174 (5)	0.0016 (5)
O1	0.0557 (6)	0.0447 (5)	0.0585 (6)	−0.0112 (4)	0.0262 (5)	−0.0036 (4)
O2	0.0796 (8)	0.0689 (7)	0.0634 (7)	0.0212 (6)	0.0021 (6)	−0.0142 (6)

### Geometric parameters (Å, °)

**Table:**

C1—N1	1.3161 (15)	C10—H10	0.9300
C1—O1	1.3667 (14)	C11—N4	1.3312 (18)
C1—C2	1.4020 (17)	C11—H11	0.9300
C2—C3	1.3744 (17)	C12—N4	1.3322 (17)
C2—H2	0.9300	C12—C13	1.3823 (17)
C3—C4	1.3928 (16)	C12—H12	0.9300
C3—H3	0.9300	C13—H13	0.9300
C4—N3	1.3842 (14)	C14—C15	1.365 (2)
C4—C5	1.3924 (15)	C14—C19	1.3708 (19)
C5—N1	1.3328 (14)	C14—O1	1.4048 (16)
C5—N2	1.3724 (14)	C15—C16	1.385 (2)
C6—N3	1.3192 (14)	C15—H15	0.9300
C6—N2	1.3788 (13)	C16—C17	1.3784 (19)
C6—C9	1.4686 (15)	C16—H16	0.9300
C7—N2	1.4638 (14)	C17—O2	1.3687 (17)
C7—C8	1.5066 (19)	C17—C18	1.371 (2)
C7—H7A	0.9700	C18—C19	1.369 (2)
C7—H7B	0.9700	C18—H18	0.9300
C8—H8A	0.9600	C19—H19	0.9300
C8—H8B	0.9600	C20—O2	1.406 (2)
C8—H8C	0.9600	C20—H20A	0.9600
C9—C13	1.3857 (17)	C20—H20B	0.9600
C9—C10	1.3899 (16)	C20—H20C	0.9600
C10—C11	1.3786 (18)		
			
N1—C1—O1	118.16 (10)	N4—C12—C13	124.07 (13)
N1—C1—C2	125.63 (11)	N4—C12—H12	118.0
O1—C1—C2	116.21 (10)	C13—C12—H12	118.0
C3—C2—C1	119.44 (11)	C12—C13—C9	118.98 (11)
C3—C2—H2	120.3	C12—C13—H13	120.5
C1—C2—H2	120.3	C9—C13—H13	120.5
C2—C3—C4	117.22 (11)	C15—C14—C19	120.64 (13)
C2—C3—H3	121.4	C15—C14—O1	119.97 (12)
C4—C3—H3	121.4	C19—C14—O1	119.35 (13)
N3—C4—C5	109.76 (10)	C14—C15—C16	119.40 (13)
N3—C4—C3	133.26 (10)	C14—C15—H15	120.3
C5—C4—C3	116.98 (10)	C16—C15—H15	120.3
N1—C5—N2	125.53 (10)	C17—C16—C15	120.04 (13)
N1—C5—C4	127.79 (10)	C17—C16—H16	120.0
N2—C5—C4	106.68 (9)	C15—C16—H16	120.0
N3—C6—N2	113.32 (9)	O2—C17—C18	115.72 (12)
N3—C6—C9	122.41 (10)	O2—C17—C16	124.63 (13)
N2—C6—C9	124.27 (10)	C18—C17—C16	119.66 (13)
N2—C7—C8	112.19 (10)	C19—C18—C17	120.31 (13)
N2—C7—H7A	109.2	C19—C18—H18	119.8
C8—C7—H7A	109.2	C17—C18—H18	119.8
N2—C7—H7B	109.2	C18—C19—C14	119.94 (13)
C8—C7—H7B	109.2	C18—C19—H19	120.0
H7A—C7—H7B	107.9	C14—C19—H19	120.0
C7—C8—H8A	109.5	O2—C20—H20A	109.5
C7—C8—H8B	109.5	O2—C20—H20B	109.5
H8A—C8—H8B	109.5	H20A—C20—H20B	109.5
C7—C8—H8C	109.5	O2—C20—H20C	109.5
H8A—C8—H8C	109.5	H20A—C20—H20C	109.5
H8B—C8—H8C	109.5	H20B—C20—H20C	109.5
C13—C9—C10	117.46 (11)	C1—N1—C5	112.91 (10)
C13—C9—C6	123.59 (10)	C5—N2—C6	105.53 (9)
C10—C9—C6	118.91 (11)	C5—N2—C7	122.62 (9)
C11—C10—C9	118.99 (12)	C6—N2—C7	131.19 (9)
C11—C10—H10	120.5	C6—N3—C4	104.72 (9)
C9—C10—H10	120.5	C11—N4—C12	116.31 (11)
N4—C11—C10	124.17 (12)	C1—O1—C14	116.08 (9)
N4—C11—H11	117.9	C17—O2—C20	117.96 (13)
C10—C11—H11	117.9		
			
N1—C1—C2—C3	−1.3 (2)	O1—C14—C19—C18	−178.17 (12)
O1—C1—C2—C3	178.21 (11)	O1—C1—N1—C5	−177.83 (11)
C1—C2—C3—C4	−0.17 (18)	C2—C1—N1—C5	1.63 (18)
C2—C3—C4—N3	−179.45 (12)	N2—C5—N1—C1	179.15 (11)
C2—C3—C4—C5	0.96 (16)	C4—C5—N1—C1	−0.73 (17)
N3—C4—C5—N1	179.77 (11)	N1—C5—N2—C6	−179.62 (11)
C3—C4—C5—N1	−0.54 (18)	C4—C5—N2—C6	0.28 (12)
N3—C4—C5—N2	−0.12 (13)	N1—C5—N2—C7	8.72 (18)
C3—C4—C5—N2	179.56 (10)	C4—C5—N2—C7	−171.38 (10)
N3—C6—C9—C13	142.45 (12)	N3—C6—N2—C5	−0.36 (13)
N2—C6—C9—C13	−37.09 (17)	C9—C6—N2—C5	179.21 (10)
N3—C6—C9—C10	−35.08 (17)	N3—C6—N2—C7	170.30 (11)
N2—C6—C9—C10	145.39 (12)	C9—C6—N2—C7	−10.13 (19)
C13—C9—C10—C11	−0.54 (19)	C8—C7—N2—C5	−76.72 (15)
C6—C9—C10—C11	177.14 (12)	C8—C7—N2—C6	113.98 (14)
C9—C10—C11—N4	1.3 (2)	N2—C6—N3—C4	0.28 (13)
N4—C12—C13—C9	1.1 (2)	C9—C6—N3—C4	−179.30 (10)
C10—C9—C13—C12	−0.60 (17)	C5—C4—N3—C6	−0.09 (13)
C6—C9—C13—C12	−178.16 (11)	C3—C4—N3—C6	−179.70 (13)
C19—C14—C15—C16	−0.1 (2)	C10—C11—N4—C12	−0.8 (2)
O1—C14—C15—C16	177.83 (11)	C13—C12—N4—C11	−0.4 (2)
C14—C15—C16—C17	0.6 (2)	N1—C1—O1—C14	−0.86 (17)
C15—C16—C17—O2	178.89 (13)	C2—C1—O1—C14	179.63 (11)
C15—C16—C17—C18	−0.7 (2)	C15—C14—O1—C1	90.64 (14)
O2—C17—C18—C19	−179.25 (14)	C19—C14—O1—C1	−91.40 (14)
C16—C17—C18—C19	0.4 (2)	C18—C17—O2—C20	−175.90 (15)
C17—C18—C19—C14	0.1 (2)	C16—C17—O2—C20	4.5 (2)
C15—C14—C19—C18	−0.2 (2)		
